# It’s not just the big kids: both high and low BMI impact bracing success for adolescent idiopathic scoliosis

**DOI:** 10.1007/s11832-016-0763-3

**Published:** 2016-08-08

**Authors:** Christine M. Goodbody, Ivor B. Asztalos, Wudbhav N. Sankar, John M. Flynn

**Affiliations:** 1 Division of Orthopaedic Surgery, 2nd Floor Wood Bldg, The Children’s Hospital of Philadelphia, 3401 Civic Center Blvd, Philadelphia, PA 19104 USA; 2 Perelman School of Medicine at the University of Pennsylvania, Philadelphia, PA USA

**Keywords:** Adolescent idiopathic scoliosis, Bracing, Body mass index, Compliance

## Abstract

**Purpose:**

Bracing is a common treatment for patients with adolescent idiopathic scoliosis (AIS) and is recommended for most skeletally immature patients with a curve of 25–45° in order to prevent or delay curve progression. The aim of this study was to determine at which body habitus orthotic management for AIS becomes less effective. We hypothesize that overweight children are more likely to fail brace treatment.

**Methods:**

This was a retrospective cohort study involving consecutive patients with AIS treated with a thoracolumbosacral orthosis at a large pediatric tertiary care center. Patients were divided into three groups based on BMI: (1) high-BMI group (BMI >85th percentile); (2) low-BMI group (BMI <20th percentile); (3) mid-BMI group (BMI 20th–85th percentile). Successful orthotic treatment was defined as an increase in the primary curve of <5°, prevention of progression past 45°, and avoidance of surgery.

**Results:**

The study cohort comprised 182 patients with a mean age of 12.5 years at brace prescription and a mean follow-up of 2 years. Compared to the mid-BMI group, high- and low-BMI patients were significantly more likely to fail orthotic management. The association between high-BMI and orthotic failure disappeared when compliance and in-brace correction were taken into account, but the association between low-BMI and each poor outcome remained significant.

**Conclusions:**

Based on our results, children on either end of the BMI spectrum are more likely to fail brace treatment for scoliosis than their mid-BMI counterparts. In high-BMI patients, this appears to be in large part attributable to an inadequacy of in-brace curve correction as well as to poorer brace compliance, while a low BMI appears to be an independent risk factor for brace failure.

*Level of evidence* III.

## Introduction

Bracing is a common treatment for patients with adolescent idiopathic scoliosis (AIS) and is often recommended for those patients with a curve of 25–45° in order to prevent or delay curve progression and the need for surgical treatment [1, 2].

There is a plethora of data supporting the efficacy of brace treatment for the prevention of scoliotic curve progression and reduction of the risk for surgical intervention, and it remains the only non-operative method proven to improve the natural history of scoliosis [3–7]. While compliance remains the main barrier to maximizing the efficacy of bracing [2, 4–6, 8, 9], other factors, such as degree of skeletal maturity, curve type, curve magnitude, ability of brace to correct curve, and, possibly, gender, may affect the risk of curve progression [2, 3, 5, 10–15]. Multiple factors associated with the type and quality of spinal orthosis used have also been shown to contribute to the amount of correction achieved. These include, but are not limited to, optimal pad placement, maximization of pad pressure and strap tension, creation of appropriate three-point pressure, including axillary extension as indicated, and force maximization at the curve apex [16–18].

Despite the proven efficacy of bracing and the expanding body of knowledge on its limitations, the effect of variations in body habitus remains poorly understood. Bracing efficacy is dependent on maximal force being transferred by the brace to the spine—force that, in theory, may be dissipated by excess truncal body fat [2, 19]. Clinical intuition has led many practitioners to believe that a larger body habitus diminishes brace efficacy, but to our knowledge only one study exists to support this claim [19]. In 2005, O’Neill and colleagues found that overweight patients were 3.1-fold more likely to experience an unsatisfactory outcome of orthotic management as compared to their normal weight counterparts [19]. To our knowledge, there are no published studies establishing whether patients with body mass indices (BMI) in the low range of the BMI spectrum have different outcomes of orthotic management of scoliosis.

The aim of this study was to re-examine the claim that high BMI negatively impacts the efficacy of bracing in AIS treatment and to update, with a new decade of data, the existing literature on this topic, as well as to further stratify patients based on BMI to determine at which body habitus orthotic management becomes less effective.

## Materials and methods

We analyzed a retrospective cohort of consecutive patients with AIS treated with a thoracolumbosacral orthosis at a single, large pediatric tertiary care center. After approval for the study had been obtained from our institutional review board, we queried our outpatient database for patients with International Classification of Diseases (ICD)-9 codes consistent with idiopathic scoliosis (737.30) who also received a brace script. Patients aged 10–17 years who presented to our institution between 1 January 2009 and 1 January 2013 and were braced with a TLSO for AIS during this period were eligible for inclusion in the study. Inclusion and exclusion criteria are detailed in Table 1.

**Table 1:** Inclusion and exclusion criteria

Inclusion criteria	Exclusion criteria
Adolescent idiopathic scoliosis diagnosis at age ≥10 years	Do not meet all inclusion criteria
Skeletal immaturity: Risser stage 0, 1, or 2	Insufficient data or follow up
Cobb angle of 25–45° at brace initiation	Prior treatment
Follow-up to:	
Skeletal maturity	
Risser stage 4 or 5	
2 years post-menarchal	
Vertical growth of <6 mm in 6 months	
*OR*	
Surgery	
*OR*	
Clinical discontinuation	

A retrospective review of medical records was performed. The data collected included patient demographics, BMI percentile, Risser stage, menarche status; radiographic curve features at brace prescription, at in-brace visit, and at final follow-up; and need for eventual surgical treatment. For patients with multiple curves, the largest curve at brace initiation was followed, as the major curve determined the indication for bracing and guided our decision-making regarding progression and/or need for surgery. A patient was counted as meeting the study endpoint of requiring surgery if the treating physician recommended a procedure be performed, regardless of whether or not the patient decided to have surgery.

Poor brace compliance was defined as having a self-reported inability to wear the brace for >12 h/day. We dichotomized around the time point of 12 h, as this is a duration of daily wear that has been consistently shown to have at least some [6]—if not maximal [5]—benefit in controlling curve progression, and as such has been frequently utilized as a cut-off in clinical research on this subject. Brace compliance was further stratified into low compliance (≤12 h/day) and non-compliance (refusal to wear brace despite physician’s recommendation).

The in-brace curve correction was assessed as the percentage curve correction, which was calculated by dividing the number of degrees of in-brace curve correction by the magnitude of the curve at brace initiation.

Patients were divided into three groups based on BMI: (1) BMI >85th percentile (high-BMI group); (2) BMI <20th percentile (low-BMI group); (3) BMI 20th–85th percentile (mid-BMI group). This study was originally intended to look at potential differential results between overweight/obese patients and non-overweight/obese patients; as such, we used the United States Centers for Disease Control and Prevention (CDC) cut-off of ≥85th percentile for BMI as overweight. However, in our initial analysis of the data, we found another inflection point at the 20th percentile, below which failure rates were also higher. Consequently, we created a third group, the low-BMI group, for analysis.

Successful orthotic treatment was defined as <5° of progression in the primary curve, prevention of progression >45°, and avoidance of surgery within the follow-up period available.

With respect to statistical analysis, all demographic and descriptive data were assessed for similarity between BMI groups by analysis of variance, with the level of significance set at *p* < 0.05. If a significant difference was found, two-group variance tests were performed between BMI cohorts to assess normality, and then either tests of proportions, the standard *t* test, or the *t* test with unequal variance was performed, as appropriate, to determine between which groups any significant difference existed. The high-BMI and low-BMI groups were independently compared by logistic regression with the mid-BMI group with regard to each outcome. Correlational analysis was performed to determine which risk factors were independently related to each outcome, and all variables of significance (*p* < 0.10), were included in a multivariate analysis.

## Results

Of the 715 patients screened, 182 met all criteria for inclusion and follow-up in this study. Of these 182 children, 26 (14.3 %) were male and 156 (85.7 %) were female. The proportion of male patients was similar in each of the BMI groups (*p* = 0.42) (Table 2) Mean age at time of brace prescription was 12.5 [standard deviation (SD) 1.4, range 10–16] years, and mean follow-up was 24 (SD 11.3, range 3–56) months (Table 2).

**Table 2:** Patient demographics

Patient demographics	Study groups based on BMI^a^		
	Mid-BMI	High-BMI	Low-BMI
Number of patients	118	29	35
Age at brace initiation (years)	12.6 (1.3)	11.9 (1.6)	12.8 (1.2)
Number of females (%)	104 (88.1)	23 (79.3)	29 (82.9)
Curve at brace initiation (°)	31.94 (5.46)	34.86 (6.36)	33.37 (5.98)

Data are presented as the mean ± standard deviation (SD) unless indicated otherwise

*BMI* Body mass index

^a^High-BMI group, BMI >85th percentile; low-BMI group, BMI <20th percentile; mid-BMI group, BMI 20th–85th percentile

Children in the high-BMI group were significantly younger at brace initiation than those in the mid-BMI group (*p* = 0.02) (Table 2). Despite this age difference, skeletal maturity, as approximated by Risser sign, was similar between all three groups at brace initiation (*p* = 0.67).

The initial curve at the start of bracing was significantly larger in the high-BMI group (*p* = 0.04) (Table 2), but this difference of < 3° was not clinically significant as the margin of error in measurement of Cobb angles is generally accepted to be ±3°. No statistically significant difference was detected between low-BMI and mid-BMI patients with respect to initial curve magnitude (*p* = 0.18).

With respect to primary curve location, 119 patients had a thoracic curve, 22 had a thoracolumbar curve, and 41 had a lumbar curve. No significant difference was found in curve location between BMI groups (*p* = 0.77).

### Curve correction

Data were available for the analysis of 166 patients with respect to in-brace curve correction (31 low-BMI patients, 110 mid-BMI patients, 25 high-BMI patients).

Patients were stratified into two groups based on the degree of in-brace correction, with good correction defined as ≥45 % correction, and poor correction defined as <45 % correction. We used the cut-off of 45 % as in our data set it proved to be an inflection point above which patients tended to be braced more successfully, and below which they were braced less successfully. As compared to the mid-BMI group, the high-BMI group had a more than fivefold higher odds of poor correction. Those in the low-BMI group showed a trend towards increased odds of poor correction, but this trend was not statistically significant (Table 3).

**Table 3:** In-brace correction by body mass index category

In-brace correction	Study groups based on BMI		
	Mid-BMI	High-BMI	Low-BMI
Average in-brace correction (%)	41.9	31.4 (*p* = 0.02)	35.6 (*p* = 0.21)
Odds for poor in-brace correction	1.0	5.5 (*p* = 0.01)	1.8 (*p* = 0.17)

Importantly, poor in-brace correction was a significant risk factor for all measures of brace failure (Table 4).

**Table 4:** Poor in-brace correction and odds ratio of poor outcome

In-brace correction	Progression of at least 5°	Progression of >45°	Need for surgery	Orthotic failure
Poor in-brace correction	3.3 (*p* < 0.01)	3.4 (*p* = 0.01)	4.0 (*p* = 0.02)	3.2 (*p* < 0.01)

### Brace compliance

Brace compliance was significantly different between BMI groups. High-BMI patients were significantly more likely to have low compliance, while low-BMI patients were significantly more likely to be grossly non-compliant (Table 5).

**Table 5:** Odds ratio of brace compliance by BMI category*

BMI groups	Low-compliance group	Non-compliance group
High-BMI	3.1 (*p* = 0.05)	1.9 (*p* = 0.18)
Low-BMI	1.3 (*p* = 0.73)	2.8 (*p* = 0.01)

* As compared to the mid-BMI group

Moreover, compliance showed a significant trend with important measures of bracing outcomes, and patients with poor compliance had significantly increased odds ratios for all measures of poor outcome (Table 6).

**Table 6:** Compliance and odds ratio of poor outcome

Compliance	Progression of at least 5°	Progression of >45°	Need for surgery	Orthotic failure
Low compliance	5.4 (*p* < 0.01)	4.0 (*p* = 0.01)	7.3 (*p* < 0.01)	5.2 (*p* < 0.01)
Non-compliance	7.1 (*p* < 0.01)	7.0 (*p* < 0.01)	10.9 (*p* < 0.01)	8.6 (*p* < 0.01)

### BMI category

The outcomes of bracing were significantly different between BMI groups. Average curve progression was 2.5 ± 8.8° in patients in the mid-BMI group compared to 5.7 ± 10.0 (*p* = 0.09) and 7.4 ± 10.9 (*p* = 0.01) in those in the high-BMI and low-BMI groups, respectively. Compared to the mid-BMI group, high-BMI patients were significantly more likely to progress at least 5°, to progress past 45°, and to experience any individual poor outcome. There was a trend towards an increased risk of surgery in this group, but this trend did not reach statistical significance. Patients in the low-BMI group were also more likely to experience curve progression and were significantly more likely to require surgical correction (Tables 7, 8).

**Table 7:** Body mass index category and poor outcome prevalence

BMI category	Progression of at least 5° (*n*) (%)	Progression of >45° (*n*) (%)	Need for surgery (*n*) (%)	Orthotic failure (*n*) (%)	Bracing success rate (%)^*^
Mid-BMI	38 (32.2)	18 (15.3)	15 (12.7)	39 (33.1)	66.9
High-BMI	16 (55.2)	11 (37.9)	7 (24.1)	16 (55.2)	44.8
Low-BMI	21 (60.0)	14 (40.0)	13 (37.1)	23 (65.7)	34.3

Data are presented as the number with the percentage in parenthesis unless indicated otherwise

* Bracing success rate = 1 − (poor outcome rate)

**Table 8:** Body mass index category and odds ratio of poor outcome according to univariate analysis*

BMI category	Progression of at least 5°	Progression of >45°	Need for surgery	Orthotic failure
High-BMI	2.6 (*p* = 0.02)	3.4 (*p* = 0.01)	2.2 (*p* = 0.13)	2.4 (*p* = 0.04)
Low-BMI	3.2 (*p* < 0.01)	3.7 (*p* < 0.01)	4.1 (*p* < 0.01)	3.7 (*p* < 0.01)

* Individual association, as compared to the mid-BMI group

### Multivariate analysis of effect of BMI on clinical outcomes

All variables determined by correlational analysis to have a possible confounding impact on the effect of BMI category on each outcome were included in the multivariate analysis for that outcome, as summarized in Table 9.

**Table 9:** Potential confounders for each outcome based on correlational analysis

Potential confounders	Progression of at least 5°	Progression of >45°	Need for surgery	Any poor outcome
Age				
Sex	X			
Curve type				
Initial Risser sign	X	X	X	X
Initial curve magnitude		X	X	
In-brace correction	X	X	X	X
Brace compliance	X	X	X	X

X designates a variable found to be significant at *p* < 0.10 in the correlational analysis

**Fig. 1:**
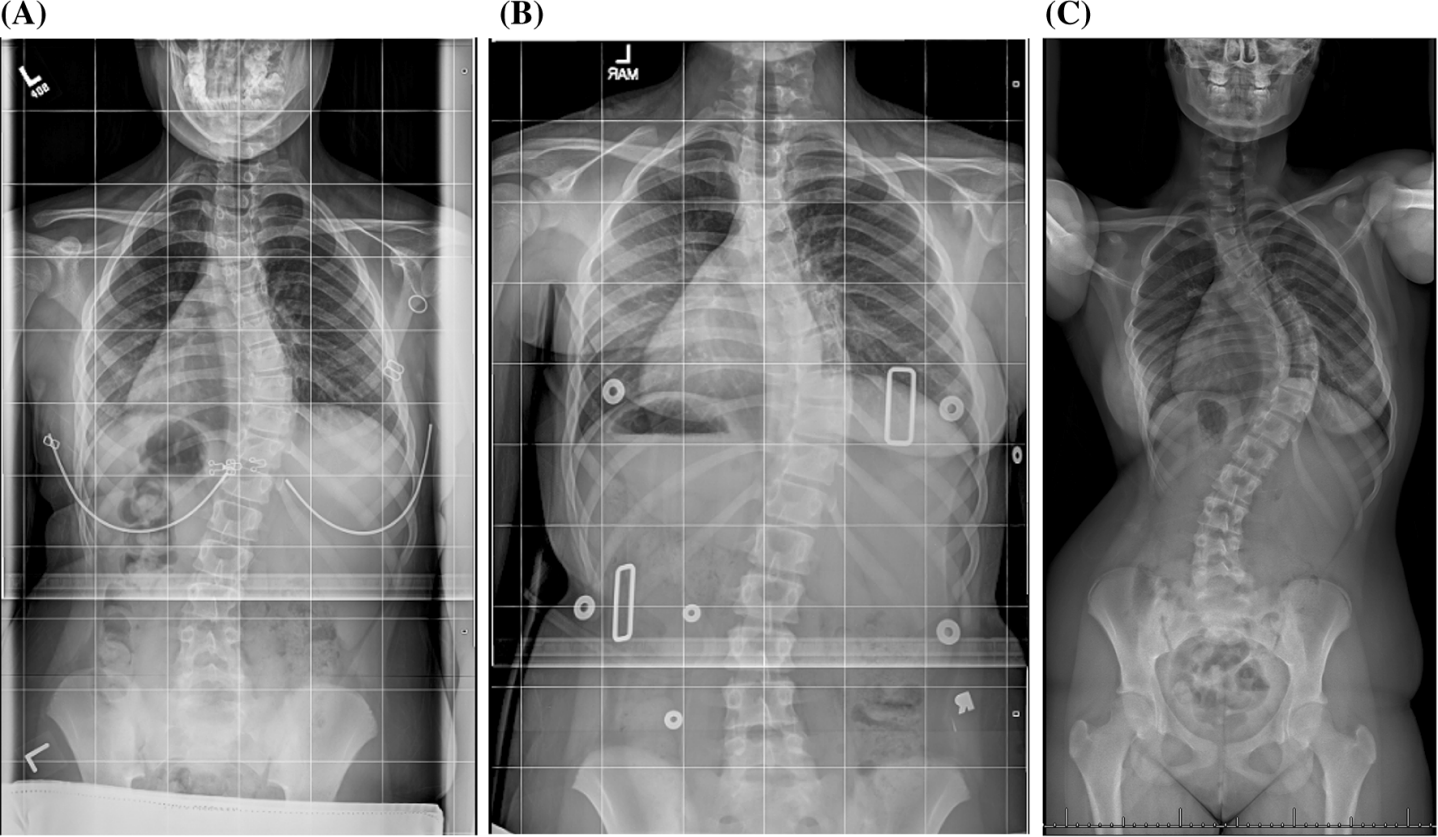
**a** A 10-year-old skeletally immature female presents with a right thoracic curve of 26° and a high body mass index (high-BMI group) at the 96.89 percentile. **b** She is prescribed a brace, and the follow-up examination reveals that her curve has been corrected to 22° (4° correction, 15.4 %), but she is noted to have low compliance with brace wear. **c** Two years after treatment initiation, her curve has progressed to 51°, and she requires surgical intervention

**Fig. 2:**
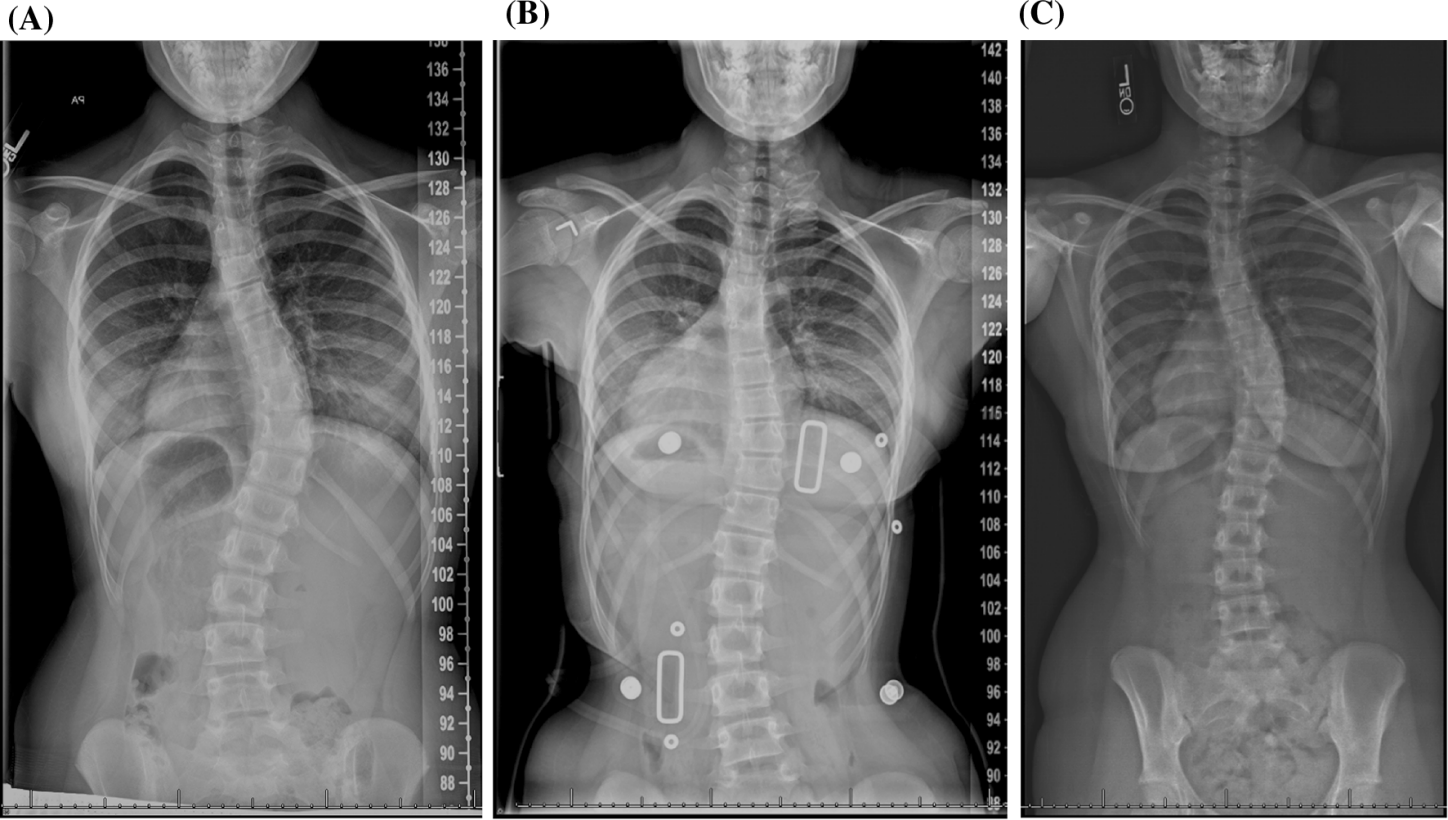
**a** A 10-year-old skeletally immature female presents with a right thoracolumbar curve of 42° and a high BMI (high-BMI group) at the 94.34 percentile. **b** She is prescribed a brace, and the follow-up examination reveals that her curve had been corrected to 16° (26° correction, 61.9 %). She is noted to have good compliance with brace wear.** c** Two years after treatment initiation, she has reached skeletal maturity and her curve, which now measures 33°, has not progressed

**Fig. 3:**
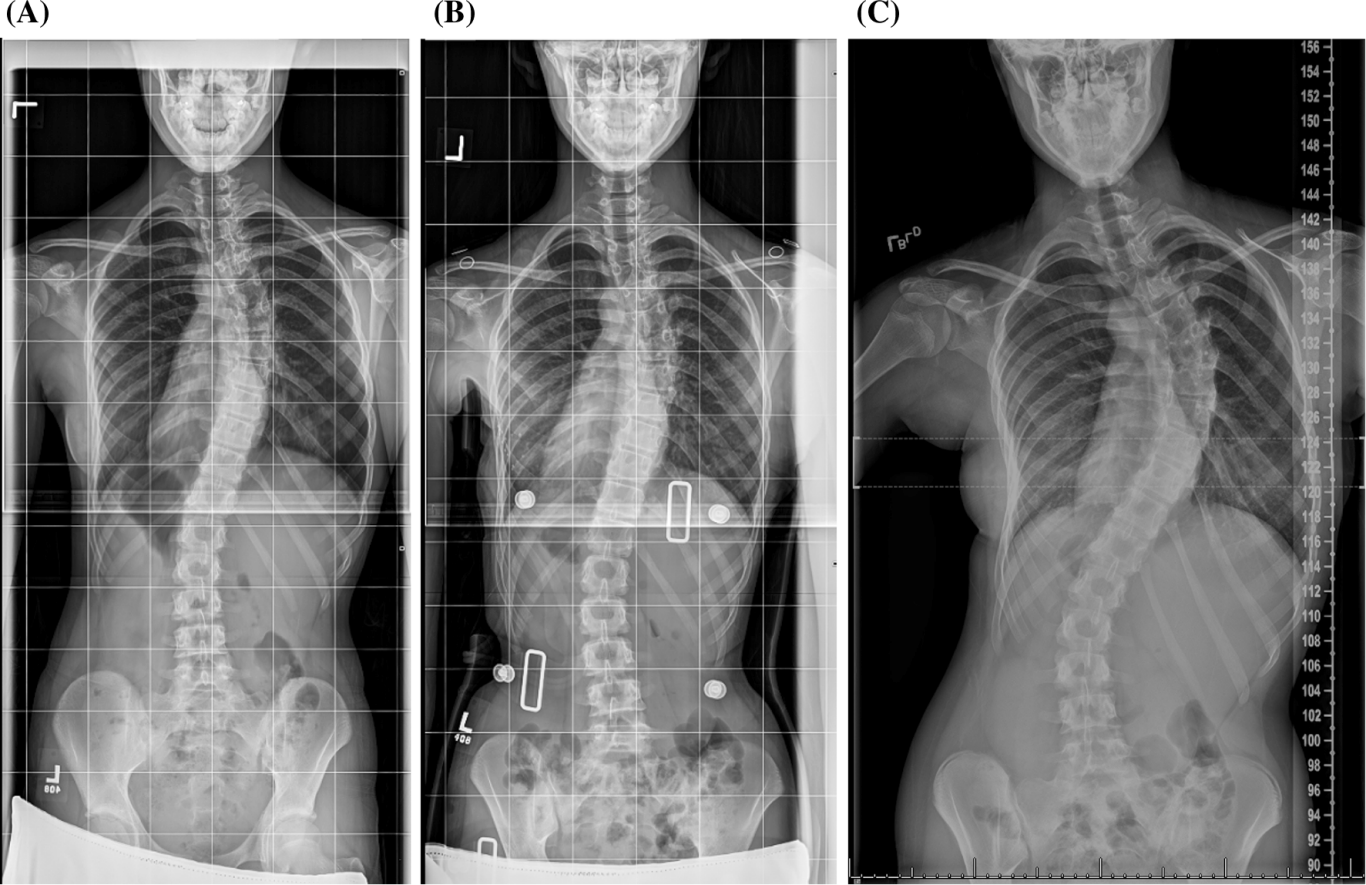
**a** An 11-year-old skeletally immature female presents with a right thoracic curve of 35° and a low BMI (low-BMI group) at the 15.05 percentile. **b** She is prescribed a brace, and the follow-up examination reveals that her curve has been corrected to 26° (9° correction, 25.7 %). She is noted to have poor compliance with brace wear. **c** One year and 7 months after treatment initiation, her curve has progressed to 60°, and she requires surgical intervention

**Fig. 4:**
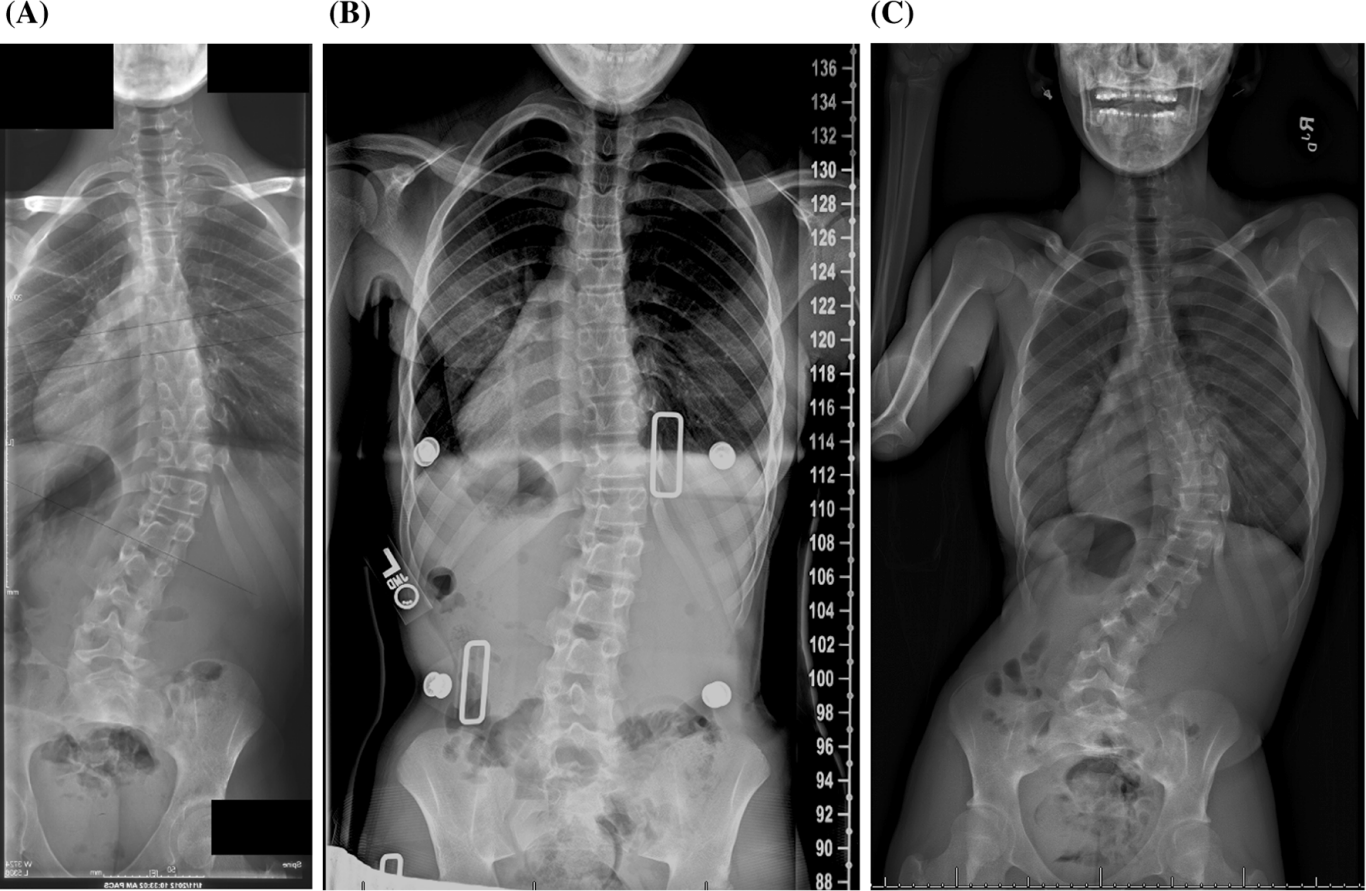
**a** A 13-year-old skeletally immature female presents with a right thoracolumbar curve of a 34° and a low BMI (low-BMI group) at the 18.56 percentile. **b** She is prescribed a brace, and the follow-up examination reveals that her curve has been corrected to 16° (18° correction, 52.9 %). She is noted to have good compliance with brace wear despite a complaint of rubbing. **b** One year and 8 months after treatment initiation, her curve has progressed to 54°, and she requires surgical intervention

With respect to all poor outcomes, when relevant confounders were controlled for, with in-brace curve correction and brace compliance always controlled for, the association between high-BMI patients and outcome risk disappeared (Figs. 1, 2). However, low-BMI patients were still significantly more likely than their mid-BMI counterparts to experience each of these poor outcomes, even when the appropriate confounding variables were accounted for (Figs. 3, 4, Table 10).

**Table 10:** Body mass index category and odds ratio of poor outcome according to multivariate analysis*

BMI category	Progression of at least 5^°^	Progression of >45°	Need for surgery	Any poor outcome
High-BMI	1.2 (*p* = 0.71)	1.3 (*p* = 0.66)	0.7 (*p* = 0.62)	1.2 (*p* = 0.70)
Low-BMI	2.8 (*p* = 0.03)	3.8 (*p* = 0.01)	3.4 (*p* = 0.03)	3.2 (*p* = 0.02)

* Accounting for all possible confounding variables, as compared to the mid-BMI group

## Discussion

It is generally accepted that the management of compliance among patients with AIS to treatment with a thoracolumbosacral orthosis can halt curve progression in the general AIS population [4–7]. However, whether or not subsets of the general AIS population are more or less amenable to successful brace treatment is still to be determined. Brace treatment for scoliosis can be prolonged and arduous and may cause anxiety and distress in patients and families alike [20–22]. Consequently, brace treatment should ideally be reserved for those patients who may reasonably expect to benefit from it. In our series, patients with a BMI falling in the high or low range of the BMI spectrum were significantly more likely to fail orthotic treatment than those with a BMI falling in the mid-range. This result confirms previous findings in children with a higher BMI [19] and, to our knowledge, is the first time children with a lower BMI have been demonstrated to have poorer outcomes as well.

The primary objective of this study was to determine whether BMI significantly impacted bracing success, and our results demonstrate that this is indeed the case. The result in the high-BMI cohort is in keeping with the findings reported by O’Neill and colleagues in 2005 [19] in the only previous study published on this topic. This study analyzed data on patients with AIS treated with a thoracolumbosacral orthosis at a large pediatric tertiary care center from 1991 to 2001, and revealed that overweight patients had a 3.1-fold greater likelihood of unsuccessful bracing than normal-weight patients. While brace wear in this study was associated with improved outcomes, in contrast to our results, it was not associated with high-BMI status. Overweight patients were found to have significantly worse in-brace correction, but the authors did not perform multivariate analysis, controlling for this and other variables correlated with outcomes, on the association between BMI and brace failure. As such, our results not only confirm the association between BMI and brace failure as detailed in this prior study, but also delineate the pathway through which these overweight and obese patients fail brace therapy—namely, through decreased compliance and poor in-brace correction.

Univariate analysis revealed that there was a significant association between the high-BMI patients and curve progression of at least 5° and progression to a curve magnitude of more than 45°, with a trend towards an association with a requirement for surgical correction. Overall, the risk of failing brace therapy was 2.4-fold higher in high-BMI patients than in mid-BMI patients. Statistically, the high-BMI group did have a slightly larger curves at treatment initiation than the mid-BMI group, although clinically the difference was not significant. This difference may be due to delayed AIS diagnosis in high-BMI patients, as the examination may be limited by body habitus, or to a reluctance by the clinician to initiate bracing in these patients.

A significant and novel finding of our study was that low-BMI patients saw an increased risk of all poor outcomes, including surgery. Compared to their mid-BMI peers, low-weight patients were 3.7-fold more likely to fail brace treatment and had a higher odds for failure than even the high-BMI group. Patients in the low-BMI cohort were the least likely to have a successful outcome, with only 34.3 % of low-BMI patients avoiding curve progression.

Our data also revealed that multiple variables, in particular in-brace correction and compliance, were correlated both with BMI and with poor outcomes. The finding that high-BMI patients were significantly more likely to have poor in-brace correction may be attributed to soft tissue interference in transmitting the corrective force of the brace to the spine. Of note, low-BMI patients showed a trend towards increased risk of poor in-brace correction as well. Hypotheses for this finding include possible decreased flexibility of curves in these patients or difficulty in tolerating tightening the brace enough to create an adequate corrective force in these more slender individuals.

In our study, high-BMI patients were more likely to demonstrate low compliance. Excess soft tissue may make the brace hotter, more restrictive, poorer fitting, and less tolerable. It is interesting that we found low-BMI patients to be significantly more likely to be completely non-compliant—that is, they more frequently reported not to wear the brace at all. Possible explanations may include increased brace discomfort caused by the brace rubbing over their more protuberant bony prominences or a possible weight-based discrepancy in tolerance of the social stress, negative cosmetic appearance, and body image issues associated with brace wear.

In addition to our results that in-brace correction and brace compliance were correlated with outcomes, our findings that multiple other variables, namely Risser sign, initial curve magnitude and, possibly, sex, may predispose to curve progression and/or brace failure, are also supported in the literature [3, 13, 15].

Multivariate analysis was performed to assess the impact of these factors, along with other possible confounding variables, on the association of BMI with brace therapy success. When these variables were accounted for, there was no longer an independent risk of failure conferred by high BMI, suggesting that most of the association of a high BMI and poor outcomes can be explained by poor curve correction and compliance. In other words, each overweight child presenting with scoliosis in the bracing range is still at an a priori higher risk for treatment failure, but if they are able to achieve adequate in-brace correction and are willing to wear the brace faithfully, the effect of their BMI can be completely mitigated and their results will be similar to that of mid-BMI children. These points must be considered when the risks and benefits of therapy are being estimated and clinical decisions are being made on how to proceed with treatment.

While low-BMI patients tended towards worse in-brace correction and were more likely to be non-compliant, the multivariate analysis revealed that these factors did not fully account for their worse outcomes. Even when all potential confounding variables were factored in, low-BMI patients were still 3.2-fold more likely than mid-BMI patients to fail brace therapy and experience a poor outcome, leading us to conclude that a low BMI is either an independent risk factor for brace failure, or it conveys risk through a variable that was not controlled for in this study. Other possible explanations for this result in low-BMI children include inaccurate or biased compliance reporting in this group (i.e. they wore the brace even less than reported), exaggerated in-brace correction due to tightening more than usual for the in-brace radiograph, and some intrinsic factor driving curve progression, such as an increased incidence of subclinical neuromuscular disease.

The limitations of this study include its retrospective nature and our inability to review a number of factors previously associated with curve progression, such as curve flexibility, due to lack of appropriate documentation. We also decided to include only patients who met Scoliosis Research Society skeletal maturity criteria for bracing (Risser sign 0, 1, 2 at brace initiation) in order to provide as homogenous a cohort as possible where most practitioners would agree on the indication for bracing. However, this will limit the generalizability of this study to Risser 3 patients, who at some institutions, including our own, may be selectively given a trial of bracing. Length of follow-up is another potential limitation of our study, as it was not designed to follow the patients up to a specific time point, but rather until skeletal maturity was reached. This choice was made because bracing is typically employed until this clinical end-point and not for a set time period. As such, variable lengths of follow-up were obtained as patients progressed to maturity at variable rates. Since we did not follow patients past skeletal maturity, we cannot guarantee that there were no cases of late progression after skeletal maturity or brace discontinuation. However, this study did follow patients sufficiently through the traditional term of brace therapy and, therefore, the duration of follow-up can be considered to be reasonable for a study evaluating curve progression during the period of brace wear. Measurement of brace compliance is another potential limitation of our study, as compliance was only noted by self-report and was dichotomized, so this data may be susceptible to reporting bias and incremental differences in brace wear could not be quantified. Additionally, it is possible that a true association between risk of surgery and a high BMI does exist, but as surgery was the least common outcome among our study participants, our study may not have been adequately powered to detect such an association. Lastly, this study was conducted at a single tertiary care institution with in-house orthotic specialists, and as such it may not be generalizable to different populations, to community-based treatment centers, or to communities where the quality of orthoses is variable.

In light of our findings, recommendations for future studies include further body habitus stratification, ideally with increased population size and power, more rigorous compliance monitoring (e.g. wear-time monitors); and reproduction, or refutation, of the effect of low-BMI on bracing outcomes, as this is a novel finding which requires validation. If our results are confirmed, further study is necessary to elucidate the cause of poorer outcomes in these lower weight children. Moreover, our study raises the question of whether psychological and/or nutritional consultation could be helpful at the initial detection of scoliosis and/or at the initiation of brace therapy, as BMI optimization is now suggested by our data to be correlated with improved bracing success.

In conclusion, we have found that both a high and low BMI correlate with failure of orthotic management for patients with AIS. The effect of high BMI on orthotic outcomes can be explained primarily by poor in-brace correction but also by low compliance. While low-BMI children saw a trend towards poorer in-brace curve correction and were more likely to be non-compliant, these variables do not fully explain their inferior results. We therefore conclude that having a low BMI is an independent risk factor for failing brace treatment. These results are useful in informing the clinical decision-making process for patients with AIS and add to the literature on the ill effects of a non-ideal BMI.
